# Lhermitte's Sign Developing after IMRT for Head and Neck Cancer

**DOI:** 10.1155/2010/907960

**Published:** 2010-06-14

**Authors:** Dong C. Lim, Patrick J. Gagnon, Sophia Meranvil, Darryl Kaurin, Linda Lipp, John M. Holland

**Affiliations:** ^1^Department of Radiation Medicine, Oregon Health & Science University, Mailcode KPV4, 3181 Southwest Sam Jackson Park Road, Portland, OR 97239-3098, USA; ^2^Tuality/OHSU Cancer Center, Hillsboro, OR 97123, USA

## Abstract

*Background*. Lhermitte's sign (LS) is a benign form of myelopathy with neck flexion producing an unpleasant electric-shock sensation radiating down the extremities. Although rare, it can occur after head and neck radiotherapy. *Results*. We report a case of Lhermitte's developing after curative intensity-modulated radiotherapy (IMRT) for a patient with locoregionally advanced oropharyngeal cancer. IMRT delivers a conformal dose of radiation in head and neck cancer resulting in a gradient of radiation dose throughout the spinal cord. Using IMRT, more dose is delivered to the anterior spinal cord than the posterior cord. *Conclusions*. Lhermitte's sign can develop after IMRT for head and neck cancer. We propose an anterior spinal cord structure, the spinothalamic tract to be the target of IMRT-caused LS.

## 1. Introduction

Lhermitte's sign (LS) was first observed in patients with multiple sclerosis by Marie and Chatelin in 1917 but it did not get world recognition in neurology until Jean Lhermitte published his report in 1920 and review in 1924 [1]. Lhermitte's sign is an electric shock-like sensation manifesting from the nape of the neck to the tips of the limbs by flexing the head forward. In general, it is a benign form of myelopathy without any permanent damage to the spinal cord. The symptoms of LS usually begin within a few months of completion of radiotherapy and are transient. This is in contrast to radiation myelitis where symptoms generally develop one year or more after radiation and progress to permanent spinal cord injury. The development of LS does not predict the development of radiation myelitis. Though LS is considered to be a classic sign of multiple sclerosis, it has been observed in patients with other conditions [2].

Lhermitte's sign has been known to be a side effect of radiotherapy (RT) of head and neck cancer patients who have received radiation to the cervical spinal cord (CSC) [3]. With the introduction of Intensity-Modulated Radiotherapy (IMRT), radiation oncologists have been able to deliver more conformal radiation to head and neck tumors. Such conformal radiotherapy should allow less radiation to the spinal cord. Although there have been cases of LS in patients with head and neck cancer receiving non-IMRT, there have been no published reports of LS in patients with head and neck cancer treated with IMRT.

We report a case of Lhermitte's sign developing in a patient with locoregionally advanced oropharyngeal squamous cell carcinoma treated successfully with concurrent chemotherapy and IMRT.

## 2. Case Report

**Figure 1:**
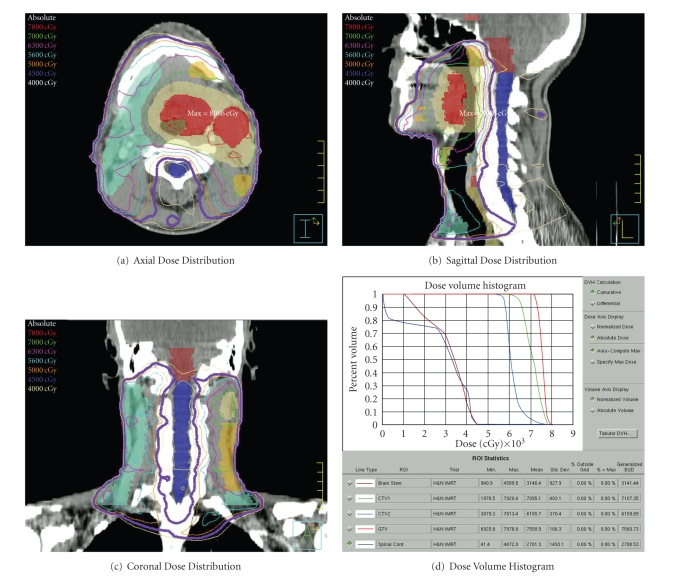
Radiation dose distribution as represented by radiation isodose lines displayed in the (a) axial, (b) sagittal, and (c) coronal planes. The thick purple line represents the 4500 cGy isodose line. The dose volume histogram (d) graphs the percent volume of structure or target receiving a radiation dose. Radiation targets including the red gross tumor volume (GTV), the beige expansion of GTV (CTV1), the yellow high-risk clinical volume (CTV2), and light blue low-risk clinical volume (CTV3) are included with the critical normal structures of spinal cord and brainstem.

Our patient is a 55-year-old gentleman with a clinical stage T2N2A moderate to poorly differentiated squamous cell carcinoma of the left tonsil. He received concurrent chemotherapy delivered during the first and fourth weeks of radiation treatment. The chemotherapy consisted of cisplatin at 100 mg/m^2^ and fluorouracil (5FU) at 1000 mg/m^2^ on days one through four. We used intensity-modulated radiotherapy with primary intent to spare the right parotid gland. A total dose of 7000 cGy was delivered to our expanded tumor target using 6 MV photons over 35 fractions of 200 cGy each. In treatment planning, the spinal cord was contoured. The maximum spinal cord point dose was 4478 cGy and the mean spinal cord dose was 2692 cGy. Figure 1 displays radiation dose distribution through isodose lines in the axial, sagittal, and coronal planes as well as the dose volume histogram (DVH) for treatment targets, the brainstem and the spinal cord. Image guidance with orthogonal pair radiographs, AP and lateral, was performed prior to each daily treatment. The patient tolerated the treatment course fairly well with expected confluent mucositis and temporary need of a feeding tube for nutrition. The patient lost 3.4 kg (4.9% of initial body weight) throughout the treatment, going from 69.2 kg to 65.9 kg at the completion of treatment on May 19, 2006; the patient showed no evidence of disease.

Four months after completion of treatment, in September 2006, the patient first developed electric-shock sensations with neck flexion causing severe right arm pain with the feeling that the extremity was swollen and numb. The severity of the pain prompted an ER visit. On the patient's followup in October, the patient had persistent difficulty with these electric-shock sensations radiating down his back into his arms with neck flexion. The patient experienced another episode in November to the left forearm requiring oxycodone to alleviate the pain. When the patient came for followup in December, he reported no further severe painful episodes but “occasional” shocks and numbness between the third and fourth fingers on both hands. In March of 2007, the patient had an “electric sensation” down his spine and into his jaw that left him with difficulty chewing for three days. At his June 2007 followup, the patient reported some mild finger numbness but no persistent electric-shock sensations. At the last followup in April 2009, the patient reported no further symptoms and was working as a contractor/remodeler. On physical examination, he had no neurologic deficits and no evidence of disease.

## 3. Discussion

Radiation to the cervical spine is a known cause of Lhermitte's sign. Leung et al. report an incidence as high as 10.3% in patients receiving non-IMRT for nasopharyngeal cancer [3]. This incidence may be higher than that seen in other head and neck sites due to the nature of radiotherapy for nasopharyngeal tumors and the close proximity of disease to the cervical spinal cord. Fein et al. report an overall incidence of 3.6% of LS in 1112 patients receiving at least 30 Gy to at least 2 cm of cervical spinal cord [4]. Chemotherapy such as cisplatin has also been reported to cause LS [5, 6], and this may have contributed to the development of LS in our case. Total radiation dose and fraction size may play a role in the risk of developing LS. Fein and colleagues describe patients receiving ≥200 cGy per fraction (one fraction per day) or ≥5000 cGy total dose to the CSC having an increased risk of developing LS. Leung finds a higher incidence of LS in patients requiring bilateral neck-boost irradiation. In these patients, the spinal cord dose was greater than 49.8 Gy and there was an 11.5% incidence of LS. This is compared with patients receiving no neck boost where the spinal cord dose was 46.8 Gy and the incidence of LS was 7.2%. However, Million and Cassisi state that total spinal cord doses as low as 3000 cGy with fractions sizes of 120 cGy may produce mild symptoms of LS [7]. With the introduction of IMRT, we have been able to deliver more conformal radiation doses to patients with head and neck cancer. We have seen 3 cases of LS develop after head and neck IMRT for 291 patients, the other two cases with much milder symptoms. Our case report patient received a cord maximum dose of 4478 cGy with 128 cGy maximum dose to the cord daily. With the conformal dose of IMRT, the mean cord dose was only 2692 cGy. In non-IMRT, opposed lateral fields are frequently utilized resulting in a much more homogeneous dose distribution to the spinal cord. Reviewing four studies where patients were treated with non-IMRT, the maximum cord dose was similar to ours at 4716 (±770 cGy) but the mean cord dose was much higher at 4026 (±425 cGy) [8–10]. Our experience of LS developing in 1% of our head and neck IMRT cases suggests that IMRT may result in a lower incidence of LS, certainly lower than the 10.3% reported by Leung and colleagues and lower than the 3.6% seen by Fein et al.

The time to development of LS in our patient of four months after chemoradiation is similar to the time to symptoms reported by Fein (mean, three months) and Leung (median, three months). Our patient's symptoms lasted approximately nine months also in line with the duration of symptoms reported by Fein (mean, six months) and Leung (median, seventeen weeks).

Jones hypothesizes that the pathophysiology of radiation-induced LS is the result of transient demyelination [9]. He believes that radiation inhibits normal proliferation of oligodendroglial cells which produce myelin. Without the myelin, the exposed sensory neurons become vulnerable to irritation from neck flexion causing electric-shock sensations. Eventually, the oligodendroglial cells recover from radiation, more myelin is produced, and the symptoms of LS abate.

**Figure 2:**
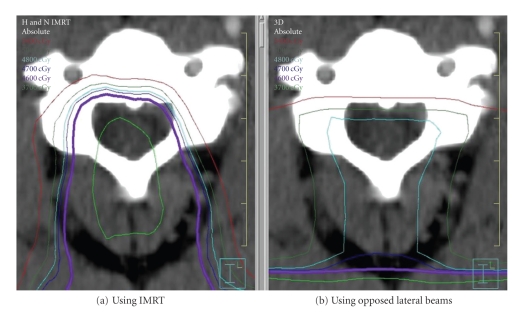
Radiation dose distribution represented by radiation isodose lines through an axial plane of the cervical spinal cord. (a) Dose distribution with IMRT and (b) dose distribution using a non-IMRT plan using opposed lateral beams. The light blue line represents the 4800 cGy isodose line and the purple line represents the 4500 cGy isodose line.

**Figure 3:**
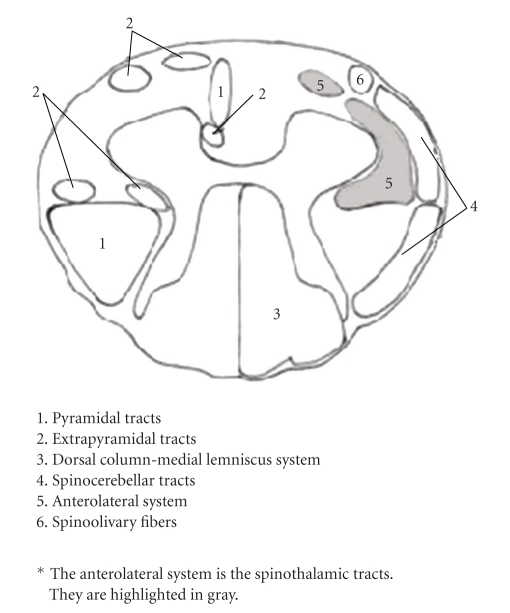
Spinal cord anatomy. The anterior spinothalamic tracts are hypothesized to be the target of IMRT-caused LS and are highlighted in gray.

Using IMRT, a much different radiation dose distribution is delivered to the spinal cord than with non-IMRT opposed lateral radiotherapy. Rather than a homogeneous dose distributed throughout the cord, there is a dose gradient with most of the radiation delivered anteriorly. Figure 2 compares the spinal cord dose distribution seen in our IMRT case with the same patient if he were treated via a non-IMRT opposed laterals technique. Butler et al. hypothesized that the dorsal columns served as the radiation target leading to LS [11]. Given the development of LS in our patient and the more anterior radiation dose distribution seen with IMRT, we suggest a more anterior spinal cord tract as the target for IMRT-caused LS. As first mentioned by Jones, we hypothesize that irradiation of the spinothalamic tract, which recognizes simple touch, pain, and temperature, is the cause of LS for our patient (Figure 3).

## 4. Conclusion

Although intensity-modulated radiotherapy delivers a much more conformal dose of radiation, its use in head and neck cancer can still lead to the development of Lhermitte's sign. Given the predominantly anterior dose distribution with IMRT, we suggest the spinothalamic tract to be the target of IMRT-caused LS.
